# The Impact of *Cryptolaemus montrouzieri* Mulsant (Coleoptera: Coccinellidae) on Control of *Dysmicoccus neobrevipes* Beardsley (Hemiptera: Pseudococcidae)

**DOI:** 10.3390/insects10050131

**Published:** 2019-05-06

**Authors:** Zhenqiang Qin, Jianhui Wu, Baoli Qiu, Shaukat Ali, Andrew G. S. Cuthbertson

**Affiliations:** 1 Sugarcane Research Institute, Guangxi Academy of Agricultural Sciences, Nanning 530007, China; 2 Department of Entomology, South China Agricultural University/Engineering Research Centre of Biological Control, Ministry of Education, Guangzhou 510640, China; jhw@scau.edu.cn (J.W.); baileyqiu@scau.edu.cn (B.Q.); aliscau@scau.edu.cn (S.A.); 3 Independent Science Advisor, York YO10 5AQ, UK; andrew_cuthbertson@live.co.uk

**Keywords:** *Cryptolaemus montrouzieri*, *Dysmicoccus neobrevipes*, efficacy, biological control

## Abstract

*Cryptolaemus montrouzieri* (Coleoptera: Coccinellidae) is an important predator of the mealybug *Dysmicoccus neobrevipes* (Hemiptera: Pseudococcidae), a major pest of *Agave sisalana* in China. Limited reports on the efficacy of *C. montrouzieri* against *D. neobrevipes* are available. This study reports the predatory efficacy and functional response of *C. montrouzieri* against *D. neobrevipes* under laboratory conditions. The prey consumption rate per day of 4th instar larvae of *C. montrouzieri* feeding on 1st instar *D. neobrevipes* nymphs (241.3 mealybugs) was the highest among the different larval life stages of the beetle. For *C. montrouzieri*, the prey consumption per day of adult females (19.8 mealybugs) was significantly higher compared to males (15.2 mealybugs) when feeding on 3rd instar *D. neobrevipes* nymphs. The functional responses of *C. montrouzieri* on 1st and 2nd instar *D. neobrevipes* nymphs were determined as Holling type II. The search rates of *C. montrouzieri* 4th instar larvae towards the 1st and 2nd instar nymphs of *D. neobrevipes* were higher than those of the other beetle life stages. In addition, the handling times of 4th instar larvae were shorter than those of the other beetle life stages. The results from this study indicate that *C. montrouzieri* can be used as a predator of *D. neobrevipes* and, therefore, it should be evaluated further for use as a biocontrol agent in *D. neobrevipes* management programs.

## 1. Introduction

The coccinellid *Cryptolaemus montrouzieri* Mulsant (Coleoptera: Coccinellidae) has been used all over the world as a biological control agent against mealybugs [1]. *C. montrouzieri,* also known as the mealybug destroyer, is a very efficient natural enemy of mealybug pests since its larvae and adult life stages both feed on the prey. It has also been successfully mass-reared within insectaries and transferred to citrus orchards and grape vineyards, as well as into glasshouses where mealybugs were a major problem [2]. In China, *C. montrouzieri* was introduced during 1955 from what was then the Soviet Union. It has been subsequently released to aid in the control of local populations of mealybugs, e.g., against *Pseudococcus* sp., a pest of *Aleurites moluccana* around the cities of Guangzhou and Foshan in Guangdong Province, China [3].

The grey pineapple mealybug, *Dysmicoccus neobrevipes* Beardsley (Hemiptera: Pseudococcidae), is an important pest of many economically important plants, such as *Agave* spp., *Cucurbita pepo* L., and *C. maxima* Duchesne [4]. Feeding by *D. neobrevipes* may cause leaf yellowing, defoliation, reduced plant growth and, in some cases, plant death. This mealybug is also a primary vector of the pineapple mealybug wilt-associated virus (PMWaV) [4]. In China, *D. neobrevipes* was first recorded in the Changjiang County, Hainan Province, where it was damaging *A. sisalana* in 1998, a major economic crop in South China [5]. Since 2006, *D. neobrevipes* has been observed on *A. sisalana* in Zhanjiang City, Guangdong Province, and its distribution has increased from 2670 ha to 6700 ha [6]. In addition, *D. neobrevipes* has recently also been observed on *A. sisalana* in the Pubei County, Guangxi Province, and on *A. sisalana*, *A. americana* var. *marginata*, and other crops in Jinghong City, Yunnan Province [7].

An important characteristic of a predator, for evaluating its impact on a given prey population, is its functional response when describing the relationship between predation and prey density [8,9,10]. A proven and ecologically sound best management practice (BMP) for *A. sisalana* is that of using biological control agents that regulate pest populations to below an economic injury level. The effects of an artificial diet containing plant pollen, population admixture, mealybug sex pheromones, and heavy metals transferred across a multitrophic food chain on the development and behaviour of *C. montrouzieri* have recently been studied [11,12,13,14]. Moreover, the structures of the sex pheromones of *D. neobrevipes* were determined and synthesised, in order to develop an efficient lure for monitoring traps [15,16]. However, there are no reports available on the efficacy of *C. montrouzieri* against *D. neobrevipes* under laboratory conditions. Therefore, in this study, we aimed to determine the functional response of *C. montrouzieri* against *D. neobrevipes*. The results are expected to provide useful reference data on this predator’s future use as a biocontrol agent for mealybug management.

## 2. Materials and Methods

### 2.1. Insects

*D. neobrevipes* was originally collected from *A. sisalana* plants at the farm of the Eastern Sisal Group Company in Zhanjiang City, Guangdong Province, China during December 2007. Individuals were maintained on ripe pumpkins under laboratory conditions (26 ± 1 °C, 75%–90% Relative Humidity, 14:10 Light:Dark photoperiod) in the Department of Entomology, South China Agricultural University, Guangzhou, China. Before starting the experiments, *D. neobrevipes* individuals were reared on *A. sisalana* plants in order to synchronise the collection of nymphs and adults of the same age.

*C. montrouzieri* individuals were maintained by feeding them on *Ferrisia virgata* Cockerell (Hemiptera: Pseudococcidae) in cages (60 cm × 60 cm × 60 cm) under laboratory conditions as outlined above. Before starting the experiments, *C. montrouzieri* individuals were reared on *D. neobrevipes* from *A. sisalana* plants for at least five consecutive generations.

### 2.2. Prey Consumption of D. neobrevipes by C. montrouzieri

Gravid females of *D. neobrevipes* were placed on fresh *A. sisalana* leaves and were allowed to produce nymphs for 2–3 days. The number of newly hatched nymphs (200 mealybugs of 1^st^ instar for the lower instar predator larvae trials and 400 mealybugs for higher predator instar larvae and adult trials) was counted and then transferred into an empty Petri dish (diameter 9 cm, height 1.5 cm). A 1st instar *C. montrouzieri* larva was placed in the Petri dish which was then sealed with preservation membranes held in place using rubber bands. Consumption of *D. neobrevipes* by *C. montrouzieri* was observed after 24 h by using a Leica M10 stereomicroscope. The predator larva was then removed and placed into another Petri dish with the same number of *D. neobrevipes* nymphs for continued observation of prey consumption. A total of 10 predator larvae were observed over two days. The consumption rates of 2nd to 4th instar larvae of *C. montrouzieri* on *D. neobrevipes* 1^st^ instar nymphs were then observed as described above. In addition, the consumption of *D. neobrevipes* 2nd and 3rd instar nymphs, immature female adults (within 3–5 days from the 3rd instar nymph to female), and mature female adults (after 20 days from the 3rd instar nymph to female) by *C. montrouzieri* 3rd and 4th instar larvae were also determined as described above.

### 2.3. Functional Response of C. montrouzieri to D. neobrevipes 

Individual *C. montrouzieri* 1st instar larvae were starved for 24 h before experiments. First instar *D. neobrevipes* nymphs were counted and placed into a Petri dish along with one *C. montrouzieri* 1st instar larva. The dish was then sealed with preservation membranes held in place with rubber bands. Consumption of the 1st instar *D. neobrevipes* nymphs by the *C. montrouzieri* 1st instar larvae was examined at five different prey densities (25, 50, 100, 200, and 400 mealybugs) after 24 h (all replicated five times) under the conditions as described for the predator consumption experiments. In addition, consumption of the 1st instar *D. neobrevipes* nymphs by *C. montrouzieri* individuals (2nd, 3rd, and 4th instar larvae, as well as female and male adults) at the five different prey densities was also determined as described above. Finally, the consumption of 2nd instar *D. neobrevipes* nymphs by *C. montrouzieri* individuals (3rd and 4th instar larvae, as well as female and male adults) at the five different prey densities was also determined as earlier described.

### 2.4. Statistical Analysis

Consumption rates per individual per day were calculated and subjected to one-way ANOVA (IBM SPSS Statistics 20, Chicago, IL, USA). Where significant, means were separated by Duncan’s method of multiple range test (*p* < 0.05).

The Holling type II functional response for *C. montrouzieri* predating on *D. neobrevipes* was fitted using the following formula [9]:

$$N_{A}=\frac{T_{t}aN_{o}}{1+abN_{o}}\;$$

where *N_A_* is the number of mealybugs eaten; *N_o_* is the density of mealybugs; *T_t_* is the total time for predators to find and deal with prey (1 day (24 h)); *a* is the searching rate; and *b* is the handling time. The searching rate (the ratio of a predator’s successful captures to contacts with prey) and handling time (including identification, capture, and consumption of prey) of *C. montrouzieri* predating on *D. neobrevipes* were fitted under the different prey densities [9]. Here, *T_t_* in this experiment is 1, therefore, the formula can then be changed to the following:

$$N_{A}=aN_{o}/(1+abN_{o})$$

The mean consumption of *D. neobrevipes* by *C. montrouzieri* at different prey densities (all replicated five times) was calculated. The mean consumption by *C. montrouzieri* of *D. neobrevipes* (*N_A_*) and the densities (*N_o_*) were fitted, and the searching rate (*a*) and handling time (*b*) were estimated (SAS 8.1 Institute, 2000, Cary, NC, USA). In addition, the correlation coefficient was analysed between the theoretical value and the experimental result with regard to the consumption of *D. neobrevipes* by *C. montrouzieri*.

## 3. Results

### 3.1. Prey Consumption of D. neobrevipes by C. montrouzieri

There were significant differences among the prey consumption rates per day of *C. montrouzieri* larvae on the 1st instar nymphs of *D. neobrevipes*, as seen in Table 1. The prey consumption per day of 4th instar larvae (241.3 mealybugs/day, SE = 19.38; *F* = 75.615; df = 3, 36; *p* < 0.001) of *C. montrouzieri* feeding on the 1st instar nymphs of *D. neobrevipes* was highest among the different larval life stages of the beetle. There were significant differences between the prey consumption per day of 3rd instar larvae and 4th instar larvae of *C. montrouzieri* feeding on the 3rd instar nymph (for 3rd instar larvae 12.2 mealybugs/day, SE = 0.88; for 4th instar larvae 23.4 mealybugs/day, SE = 1.83; *F* = 30.028; df = 1, 18; *p* < 0.001) and mature adults (for 3rd instar larvae 0.7 mealybugs/day, SE = 0.20; for 4th instar larvae 1.6 mealybugs/day, SE = 0.35; *F* = 5.631; df = 1, 18; *p =* 0.029) of *D. neobrevipes*. The prey consumption per day of 3rd and 4th instar larvae of *C. montrouzieri* feeding on the 2nd instar nymph (for 3rd instar larvae 41.6 mealybugs/day, SE = 2.49; for 4th instar larvae 50.1 mealybugs/day, SE = 3.82; *F* = 3.515; df = 1, 18; *p =* 0.077) and immature adults (for 3rd instar larvae 6.2 mealybugs/day, SE = 0.27; for 4th instar larvae 9.3 mealybugs/day, SE = 1.68; *F* = 3.305; df = 1, 18; *p =* 0.086) of *D. neobrevipes* were similar to each other. There were significant differences among the prey consumption rates per day of *C. montrouzieri* 3rd and 4th instar larvae on the 1st and 2nd instar nymphs of *D. neobrevipes* compared to the other life stages tested in this study, and no significant differences on 3rd instar nymphs, immature or mature mealybug adults. In addition, there was a significant difference between the prey consumption rates per day of female and male adults of *C. montrouzieri* feeding on the 3rd instar nymphs (for female adults 19.8 mealybugs/day, SE = 1.21; for males 15.2 mealybugs/day, SE = 1.46; *F* = 5.877; df = 1, 18; *p =* 0.026) of *D. neobrevipes*. The results from the study showed that there were significant differences among the prey consumption rates per day of *C. montrouzieri* female adults on the 1st and 2nd instar nymphs of *D. neobrevipes* compared with the other life stages tested, and no significant differences between 3rd instar nymphs with immature adults and mature adults. In turn, for *C. montrouzieri* male adults, there were significant differences among the prey consumption rates per day of the predator on the 1st and 2nd instar nymphs of *D. neobrevipes* compared with the other life stages tested, and no significant differences on 3rd instar nymphs, immature and mature mealybug adults, as seen in Table 2.

### 3.2. Functional Response of C. montrouzieri to D. neobrevipes

The functional responses of *C. montrouzieri* predating on *D. neobrevipes* were determined as Holling type II. The search rate of *C. montrouzieri* 4th instar larvae (1.0716) feeding on the 1st instar nymphs of *D. neobrevipes* was higher than those of the predator on other life stages, whereas the handling time of 4th instar larvae (0.00123) was shorter than that of the predator for other life stages. When preying on *D. neobrevipes* 2nd instar nymphs, the search rate of *C. montrouzieri* 4th instar larvae (1.0124) was also higher than that of other life stages and, in addition, the handling time of 4th instar larvae (0.0108) was shorter than that of other life stages of the predator, as seen in Table 3.

## 4. Discussion

Adult mealybugs are difficult to control because of the thick waxy secretions they produce that surround their body. As a result, repeated application of chemicals targeting immatures have usually been applied to suppress their populations [17]. However, the current trend is to control the mealybugs by using biological controls. Natural predators are now the important biological agents being used for mealybug management in many countries. The reduction rate reached 100% for all life stages of *Planococcus citri* on *Codiaeum variegatum* after three months following the release of *C. montrouzieri* in Egypt [18]. The predatory coccinellid *C. montrouzieri* is considered as one of the most popular biological agents used for mealybug control [19,20,21]. Across the world, *C. montrouzieri* has been periodically introduced by means of augmentative releases against many species of mealybugs [2,22,23,24,25]. In China, *C. montrouzieri* is used as an effective natural enemy of mealybugs [3,26,27]. It has also been demonstrated that *C. montrouzieri* can complete its development (the development period of egg to adult is 29.3 ± 1.19 days) and reproduce (38.9 ± 4.43 eggs) when feeding on *D. neobrevipes*, and its finite rate of increase is 0.0546 [28].

Many experiments have been conducted under laboratory conditions to measure the functional responses of predator coccinellids against psyllids, aphids, and mealybugs [25,29,30,31]. The results from the current study will now provide additional useful reference data for consumption of *D. neobrevipes* by *C. montrouzieri* under laboratory conditions. The prey consumption of 4th instar larvae of *C. montrouzieri* on the 1st instar nymphs of *D. neobrevipes* were higher than all the other larval life stages of the predator. The functional responses of *C. montrouzieri* on the 1st and 2nd instar nymphs of *D. neobrevipes* were determined as Holling type II. Handling times decreased from the younger to older larvae of *C. montrouzieri* as expected, due to the higher consumption of prey, which is similar to the findings of Papanikolaou et al. [30]. The authors showed that all larval instars of *Propylea quatuordecimpunctata* (L.) exhibited a Holling type II functional response when searching for *Aphis fabae* Scopoli on *Vicia faba* plants [30]. Similarly, in the study by Qin et al. [27] it was shown that there was a significant difference between the average consumption per day of the 1st instar *Saccharicoccus sacchari* nymph by female and male adults of *C. montrouzieri* with 324.35 and 303.10 mealybugs consumed per day, respectively. The functional responses of the female and male adults of *C. montrouzieri* on *S. sacchari* nymphs were also determined as Holling type II [27]. 

In the Holling type II functional response, prey consumption increases asymptotically to a plateau with increasing prey density [30]. Functional response curves are used to understand the basic mechanisms that drive the interactions of predator–prey behaviour, to clarify co-evolutionary relationships and to improve practical predictive powers for biological control [32,33]. Although there are various types of functional responses described for coccinellids [34], type II is the most common [30]. That is, the number of discs removed from the table increase at a decreasing rate until the curves level off [9]. The effect of the density of *A. fabae* on each larval instar of *P. quatuordecimpunctata* showed that the coccinellid caused an inverse density-dependent mortality of its aphid prey [30]. Work by Silva et al. [29] showed that the prey host plants can affect the consumption rates of a predator at different densities under laboratory conditions, perhaps showing the need to involve the integration of biological control with host-plant resistance. A predator can also respond to the pheromone stimuli of its prey and can do this in a species-specific manner [14]. However, functional response experiments conducted under laboratory conditions may not be representative of field conditions [30]. The feeding potential is of great interest particularly with regard to predatory insects, where their ability in consuming prey is extremely vital for their success in biological control programs. Generally, in many biocontrol programs, augmentation of natural enemies is followed by either inoculative or inundative releases to achieve satisfactory control of target pests [35]. *C. montrouzieri,* as a natural predator used to control mealybugs [36], is considered safe to humans, a good alternative to conventional pesticides, amenable to small-scale local production and addresses increased public awareness of environmental concerns [20]. Therefore, the results of the present study provide a basic understanding of *C. montrouzieri*–prey interactions. However, *C. montrouzieri* should be further evaluated as a biological control agent in *D. neobrevipes* management programs in the field.

## 5. Conclusions

The results from this study indicate that *C. montrouzieri* can be used as a predator of *D. neobrevipes* and, therefore, it should be evaluated further for possible use as a biocontrol agent in *D. neobrevipes* management programs.

## Figures and Tables

**Table 1:** Prey consumption rates per day (mean ± standard error) for different larval stages of *Cryptolaemus montrouzieri* on different life stages of *Dysmicoccus neobrevipes.*

*C. montrouzieri*	1st instar nymph	2nd instar nymph	3rd instar nymph	*D. neobrevipes*				
				Immature Adult	Mature Adult	*F*	df	*Pr* > *F*
1st instar larvae	20.1 ± 2.92 d	-	-	-	-			
2nd instar larvae	66.1 ± 5.35 c	-	-	-	-			
3rd instar larvae	108.9 ± 8.21 b A	41.6 ± 2.49 a B	12.2 ± 0.88 b C	6.2 ± 0.27 a C	0.7 ± 0.20 b C	134.689	4, 45	0.000
4th instar larvae	241.3 ± 19.38 a A	50.1 ± 3.82 a B	23.4 ± 1.83 a C	9.3 ± 1.68 a C	1.6 ± 0.35 a C	126.532	4, 45	0.000
*F*	75.615	3.515	30.028	3.305	5.631			
df	3, 36	1, 18	1, 18	1, 18	1, 18			
*Pr* > *F*	0.000	0.077	0.000	0.086	0.029			

Means in columns followed by different small letters are significantly different among the prey consumption rates per day for different larval stages of *C. montrouzieri* on the same life stages of *D. neobrevipes* (*p <* 0.05) according to Duncan’s multiple range test, and different capital letters are significantly different among the prey consumption rates per day for 3rd and 4th larvae of *C. montrouzieri* on different life stages tested of *D. neobrevipes* (*p <* 0.05) according to Duncan’s multiple range test.

**Table 2:** Prey consumption rates (mean ± standard error) per day for adult *Cryptolaemus montrouzieri* on different life stages of *Dysmicoccus neobrevipes.*

*C. montrouzieri*	1st instar nymph	2nd instar nymph	3rd instar nymph	*D. neobrevipes*				
				Immature Adult	Mature Adult	*F*	df	*Pr* > *F*
Female adult	95.8 ± 6.89 a A	56.2 ± 5.48 a B	19.8 ± 1.21 a C	11.8 ± 0.73 a CD	1.1 ± 0.05 a D	95.015	4, 45	0.000
Male adult	86.9 ± 11.21 a A	47.6 ± 6.82 a B	15.2 ± 1.46 b C	10.4 ± 1.02 a C	1.3 ± 0.13 a C	35.290	4, 45	0.000
*F*	0.458	0.966	5.877	1.368	1.433			
df	1, 18	1, 18	1, 18	1, 18	1, 18			
*Pr* > *F*	0.507	0.339	0.026	0.257	0.247			

Means in columns followed by different small letters are significantly different among the prey consumption rates per day for female and male adults of *C. montrouzieri* on the same life stages of *D. neobrevipes* (*p <* 0.05) according to Duncan’s multiple range test, and different capital letters are significantly different among the prey consumption rates per day for female and male adults of *C. montrouzieri* on different life stages tested of *D. neobrevipes* (*p <* 0.05) according to Duncan’s multiple range test.

**Table 3:** Functional response parameters and Holling type II model of *Cryptolaemus montrouzieri* life stages to 1st and 2nd instar nymphs of *Dysmicoccus neobrevipes.*

*C. montrouzieri*	*D. neobrevipes*	Search Rate	Handling Times	Correlation Coefficient	Holling Type II Model
1st instar larvae	1st instar nymphs	0.8217	0.0117	0.9944	*N_A_* = 0.8217 *N_o_*/(1 + 0.00961389 *N_o_*)
2nd instar larvae		0.8899	0.00859	0.9878	*N_A_* = 0.8899 *N_o_*/(1 + 0.007644241 *N_o_*)
3rd instar larvae		0.9314	0.00347	0.9962	*N_A_* = 0.9314 *N_o_*/(1 + 0.003231958 *N_o_*)
4th instar larvae		1.0716	0.00123	0.9994	*N_A_* = 1.0716 *N_o_*/(1 + 0.001318 *N_o_*)
Female adult		0.9257	0.00648	0.9814	*N_A_* = 0.9257 *N_o_*/(1 + 0.005998536 *N_o_*)
Male adult		0.9193	0.00772	0.9966	*N_A_* = 0.9193 *N_o_*/(1 + 0.007096996 *N_o_*)
3rd instar larvae	2nd instar nymphs	0.9349	0.0122	0.9991	*N_A_* = 0.9349 *N_o_*/(1 + 0.01140578 *N_o_*)
4th instar larvae		1.0124	0.0108	0.9834	*N_A_* = 1.0124 *N_o_*/(1 + 0.010934 *N_o_*)
Female adult		0.9373	0.0121	0.9816	*N_A_* = 0.9373 *N_o_*/(1 + 0.01134133 *N_o_*)
Male adult		0.9287	0.0127	0.9828	*N_A_* = 0.9287 *N_o_*/(1 + 0.01179449 *N_o_*)

Note: *N_A_* is the consumption by the predator *C. montrouzieri* of *D. neobrevipes*; *N_o_* is prey density.
